# Post-COVID-19 Seasonality of Influenza, Respiratory Syncytial Virus, and SARS-CoV-2 Among Hospitalized Children in Western Iran: A Molecular Surveillance Study (2023–2024)

**DOI:** 10.1007/s44197-025-00497-5

**Published:** 2025-12-09

**Authors:** Ensieh Masoorian, Ali Teimoori, Somaye Bakhtiari, Farid Azizi Jalilian, Roya Najafi Vosough, Nastaran Ansari

**Affiliations:** 1 Department of Medical Virology, Faculty of Medicine, Hamadan University of Medical Sciences, Fahmideh Street, Hamadan, Iran; 2 Reference Laboratory of Public Health, Hamadan University of Medical Sciences, Fahmideh Street, Hamadan, Iran; 3 Molecular Medicine Research Center, Hamadan University of Medical Sciences, Hamadan, Iran; 4 Research Center for Health Sciences, Hamadan University of Medical Sciences, Hamadan, Iran

**Keywords:** Respiratory syncytial virus (RSV), Influenza A and B, SARS-CoV-2, Pediatric hospitalization, Post-COVID-19 surveillance, Epidemiology, Seasonality, Iran

## Abstract

**Background:**

This study aimed to characterize the incidence, seasonality, and co-infection patterns of respiratory syncytial virus (RSV), influenza A and B, and SARS-CoV-2 among hospitalized children aged 0–5 years in Hamedan Province, a semi-arid region in western Iran, from April 2023 to March 2024. Key research questions included assessing post-pandemic shifts in viral seasonality, evaluating the extent of RSV circulation, and determining the frequency of co-infections in a resource-limited pediatric setting where regional data remain scarce.

**Methods:**

A total of 586 nasopharyngeal/oropharyngeal samples were collected from children aged 0–5 years hospitalized with acute respiratory symptoms (≥ 2 of: fever ≥ 38 °C, cough, dyspnea, oxygen saturation < 95%). Multiplex real-time PCR (sensitivity 95%, specificity 98%) was used to detect RSV, SARS-CoV-2, and influenza A (H1N1, H3N2) and B. Statistical analysis included chi-square and Fisher’s exact tests, and generalized linear models (binomial distribution, logit link).

**Results:**

Among 586 inpatients (mean age: 2.8 years; 62.5% male), 27.0% tested positive for influenza (60% influenza A [35% H1N1, 25% H3N2], 40% influenza B), 6.0% for RSV, and 6.3% for SARS-CoV-2. Influenza peaked in autumn (41.3%, p < 0.001), RSV in winter (18.2%, p < 0.001), and SARS-CoV-2 in spring (15.3%, p = 0.005). Co-infections were rare (0.9%).

**Conclusions:**

Findings reveal altered post-pandemic seasonality, reduced RSV activity, and low co-infection rates, suggesting potential ecological and immunological shifts. These trends highlight the need for sustained virus-specific surveillance and recalibrated vaccination strategies—particularly influenza vaccination in autumn and RSV prophylaxis in winter—in resource-limited pediatric settings.

## Background

Acute respiratory infections (ARIs), predominantly driven by viruses such as respiratory syncytial virus (RSV), influenza, and rhinoviruses, remain a leading cause of hospitalization and mortality among children under five years of age. This burden is especially severe in low- and middle-income countries (LMICs), where healthcare systems often face substantial resource constraints [1, 2]. Prior to the COVID-19 pandemic, ARIs accounted for approximately 15% of global pediatric deaths [3]. RSV alone was responsible for over 75,000 deaths and 24.8 million infections annually, placing a considerable burden on public health systems worldwide [4].

The emergence of SARS-CoV-2 and the global implementation of non-pharmaceutical interventions (NPIs)—including school closures, mask mandates, and travel restrictions—led to unprecedented disruptions in the transmission dynamics of common pediatric respiratory viruses [5, 6]. These measures resulted in the temporary suppression of RSV, influenza, and other respiratory pathogens during the early pandemic period, significantly altering previously well-characterized seasonal circulation patterns [7, 8]. Following the relaxation of NPIs, however, atypical and off-season resurgences of respiratory viruses were documented across multiple regions [9, 10], raising concerns about population-level immunity gaps due to reduced exposure and the long-term effects of altered viral ecology [11, 12].

For example, in southwestern Iran, RSV reappeared in spring 2022 after a near-total absence during the height of the pandemic [11]. Similarly, countries such as Mexico, Saudi Arabia, Canada, China, and Argentina reported increased pediatric hospitalizations related to RSV, influenza, and parainfluenza—often occurring outside their historical seasonal peaks [12–16]. These trends suggest a global reset of viral transmission patterns; however, robust, longitudinal surveillance data from Middle Eastern countries [17]—including Iran—remain limited. This is particularly concerning given that regional variations in demographic structure, climatic conditions, and healthcare access may uniquely influence viral transmission and resurgence.

Understanding the current seasonality and incidence of respiratory viruses is critical for outbreak prediction, targeted vaccination strategies (e.g., administering influenza vaccines in autumn and RSV prophylaxis in winter), and optimal allocation of healthcare resources—particularly in LMICs where pediatric populations play a central role in virus transmission within households and communities. In Iran, pandemic-related disruptions in virological monitoring and a research focus on COVID-19–specific outcomes, such as multisystem inflammatory syndrome in children (MIS-C), have limited the availability of comprehensive post-pandemic epidemiological data on other major respiratory pathogens [18, 19].

This study aims to systematically characterize the incidence, seasonal distribution, and co-circulation of RSV, influenza, and SARS-CoV-2 among hospitalized children aged 0–5 years in Hamedan Province, western Iran, during the 2023–2024 post-COVID-19 surveillance period. Using multiplex PCR diagnostics conducted across all counties in the province, this investigation constitutes the first post-pandemic, province-wide molecular surveillance effort of its kind in western Iran. To our knowledge, it is also the first large-scale study in Iran to concurrently assess these three respiratory viruses in hospitalized young children in the post-COVID-19 era. Given Hamedan’s cold, semi-arid climate and its representativeness of much of the western Iranian plateau, the findings from this study offer actionable insights for optimizing public health strategies, including vaccination and surveillance policies, in Iran and comparable resource-limited settings.

## Materials and methods

### Study Design and Population

This cross-sectional, province-wide surveillance study was conducted from April 2023 to March 2024 in Hamedan Province, western Iran. Respiratory specimens were collected from 586 inpatient children aged 0–5 years presenting with acute respiratory symptoms (≥ 2 of: fever ≥ 38 °C, cough, dyspnea, oxygen saturation < 95%) at various hospitals across Hamedan Province. The minimum required sample size was calculated using the single-proportion formula $\:n=\frac{{z}^{2}\times\:P\times\:(1-P)}{{d}^{2}}$, with expected proportions derived from recent Iranian pediatric studies [25]. Based on the highest requirement (≈ 544 for SARS-CoV-2, assuming *P* = 15% and d = 0.03), we recruited 586 children to ensure adequate statistical power for all target viruses. All samples were sent to the Reference Health Laboratory of Hamedan Province for analysis. Inclusion criteria required hospitalization for acute respiratory symptoms, with no hospitalization in the prior 14 days to minimize nosocomial transmission bias. Children with chronic respiratory conditions or recent antibiotic use were excluded. The study was approved by the Ethics Committee of Hamedan University of Medical Sciences (approval code: IR.UMSHA.REC.1403.807), with written informed consent obtained from parents or guardians.

### Sample Collection and Transport

Nasopharyngeal and oropharyngeal swabs were collected using sterile Dacron swabs (Good Care, China) and placed in 4 mL of viral transport medium (VTM). Samples were transported to the virology laboratory under refrigerated conditions (2–8 °C) using cold chain packaging to ensure RNA integrity. Sample quality was verified by assessing RNA concentration and purity via spectrophotometry (A260/A280 ratio ≥ 1.8).

### Molecular Detection of Respiratory Viruses

Viral RNA was extracted using the Beh Gen extraction kit (BPVD050, Iran) according to the manufacturer’s protocol. Real-time multiplex PCR was performed using the Geneova diagnostic kit (GA-SARSFluASV.100, Iran; sensitivity 95%, specificity 98%) to detect respiratory syncytial virus (RSV), SARS-CoV-2, and influenza A and B. Influenza A-positive samples were subtyped into H1N1 and H3N2 using gene-specific probes. Primers and probes are detailed in Tables 1 and 2, including sequences and annealing temperatures. Amplification was conducted on a Rotor-Gene Q thermocycler (QIAGEN), with a cycle threshold (Ct) of < 35 defining positivity for virus-specific amplification curves, as validated by the kit manufacturer’s guidelines. Internal controls, including amplification of the human RNase P gene, were used to verify sample integrity and assay reliability.

**Table 1:** Primers and probes for detection of influenza A and B

Primer/probes	Sequences 5^,^ to 3^,^
InfA Forward	GAC CRA TCC TGT CAC CTC TGA C
InfA Reverse	AGG GCA TTY TGG ACA AAK CGT CTA
InfA probe	TGC AGT CCT CGC TCA CTG GGC ACG
InfB Forward	GAG ACA CAA TTG CCT ACC TGC TT
InfB Reverse	TTC TTT CCC ACC GAA CCA AC
InfB probe	AGA AGA TGG AGA AGG CAA AGC AGA ACT AGC

† *Abbreviations*: *InfA* influenza A, *InfB* influenza B. Primer and probe sequences were provided by the World Health Organization (WHO) protocols for influenza typing and are subject to periodic updates by WHO. Samples were tested using a Rotor-Gene Q thermocycler with a cycle threshold (Ct) <40 for positivity

**Table 2:** Primers and probes to distinguish between different subtypes of influenza A

Primer/probes	Sequences 5^,^ to 3^,^
InfA Forward SW	GCA CGG TCA GCA CTT ATY CTR AG
InfA Reverse SW	GTG RGC TGG GTT TTC ATT TGG TC
SW InfA probe	6-FAM-CYA CTG CAA GCC CAT ACA CAC AAG CAG GCA-BHQ-1
AH_3_ Forward	AAG CAT TCC YAA TGA CAA ACC
AH_3_ Reverse	ATT GCR CCR AAT ATG CCT CTA GT
AH_3_ probe	6-FAM-CAG GAT CAC ATA TGG GSC CTG TCC CAG- BHQ-1
H_1_ Forward	AAA CTA TGC AAA CTA AGA GGG CT
H_1_ Reverse	TGT TTC CAC AAT GTA GGA CCA
H_1_ probe	6-FAM- CCA GAG TGT GAA TCA CTC TCC ACA-BHQ-1

† *Abbreviations*: SW, Swine influenza A (H1N1)-related genes; H1, influenza A subtype H1N1; AH3, influenza A subtype H3N2. Primer and probe sequences targeting hemagglutinin (HA) and neuraminidase (NA) genes were provided by the World Health Organization (WHO) protocols for influenza A subtyping and are subject to periodic updates by WHO. Subtyping was performed using a Rotor-Gene Q thermocycler with a cycle threshold (Ct)<40 for positivity

### Data Management and Statistical Analysis

Demographic, clinical, and molecular data were recorded in a centralized electronic database and analyzed using SPSS v24 (IBM Corp., Armonk, NY) and GraphPad Prism v9. Visualizations were created using Seaborn v0.11 and Matplotlib v3.5 in Python, with Fisher’s exact tests performed using SciPy v1.8. Descriptive statistics summarized demographics, seasonal trends, and virus-specific positivity rates, with categorical variables reported as frequencies (%) and continuous variables as means (SD). Chi-square tests assessed differences in virus positivity by sex and season. Fisher’s exact tests were used when expected cell counts were < 5. Logistic regression models evaluated associations between virus positivity and demographic factors (age, sex, geographic region). Seasonal trends were analyzed using generalized linear models (GLMs) with a binomial distribution and logit link to model virus positivity. Both logistic regression and GLM represent multivariate approaches, allowing us to assess observed associations and seasonal effects while adjusting for potential confounders, rather than relying solely on univariate comparisons. To ensure that seasonal comparisons were not limited to descriptive visualization, virus-specific positivity rates were first calculated for each season (spring, summer, autumn, winter), and formal hypothesis testing (Chi-square/Fisher) together with regression modeling (GLM) was applied to confirm statistically significant seasonal differences. A significance level of *p* < 0.05 was applied, with 95% confidence intervals (CIs) reported for incidence estimates.

## Results

From April 2023 to March 2024, 586 respiratory specimens were collected from inpatient children aged 0–5 years (mean age: 2.8 years, SD: 1.4) presenting with acute respiratory symptoms in Hamedan Province, Iran. The study population comprised 366 males (*n* = 366; 62.5%) and 220 females (*n* = 220; 37.5%). Geographically, 255 participants (*n* = 255; 43.5%) resided in Hamedan City, and 331 (*n* = 331; 56.5%) were from other provincial regions. Sample collection was highest in autumn (*n* = 264; 45.0%), followed by winter (*n* = 187; 31.9%), spring (*n* = 111; 18.9%), and summer (*n* = 24; 4.1%), as shown in Table 3.

**Table 3:** Demographic description of the patients

Variable	Category	Number of cases	Percentage (%)
Sex	Female	220	37.5
	Male	366	62.5
Case classification	Inpatient	586	100
	Outpatient	0	0
Region	Hamedan	255	44.0
	Other regions	331	56.0
Season	Winter	187	31.9
	Spring	111	18.9
	Summer	24	4.1
	Autumn	264	45.0

† Data represent 586 children aged 0–5 years hospitalized with acute respiratory symptoms. Percentages are calculated based on the total sample size (*n*=586) unless otherwise specified

**Fig. 1:**
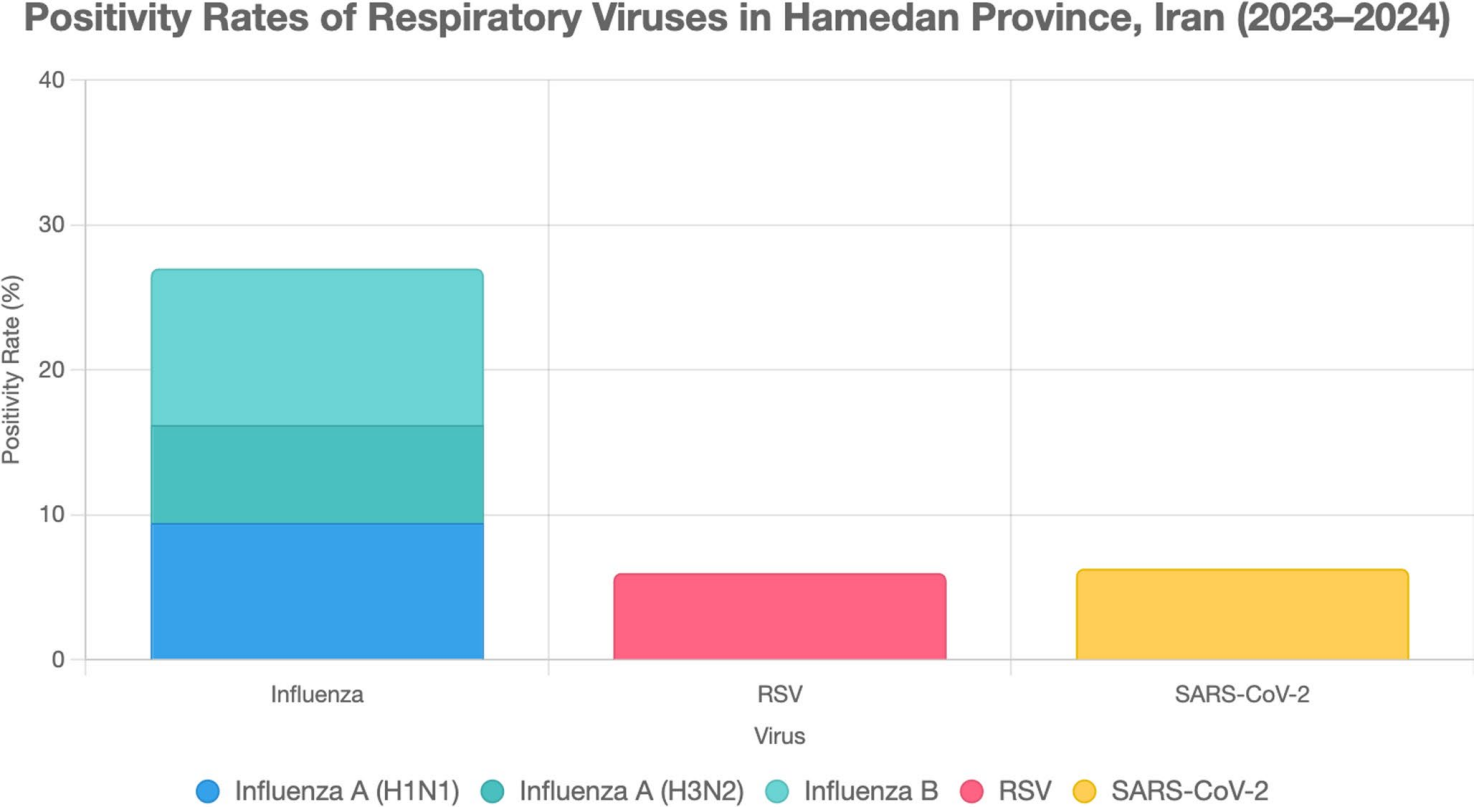
Positivity Rates of Respiratory Viruses in Hamedan Province, Iran (2023–2024). Stacked bar chart showing the percentage of inpatient children aged 0–5 years (*n* = 586) testing positive for influenza (27.0%, *n* = 158; H1N1: 35%, *n* = 55; H3N2: 25%, *n* = 40; influenza B: 40%, *n* = 63), RSV (6.0%, *n* = 35), and SARS-CoV-2 (6.3%, *n* = 37) via multiplex real-time PCR. Error bars represent 95% confidence intervals for overall positivity rates. Sample sizes (n) are annotated above each bar or segment

Multiplex real-time PCR identified 158 children (*n* = 158; 27.0%, 95% CI: 23.4–30.8) who tested positive for influenza, 37 (*n* = 37; 6.3%, 95% CI: 4.5–8.6) for SARS-CoV-2, and 35 (*n* = 35; 6.0%, 95% CI: 4.2–8.2) for RSV. Among influenza cases, 60% were influenza A (H1N1: 35%, H3N2: 25%) and 40% were influenza B (Fig. 1). Co-infections were rare (*n* = 5; 0.9%), with two cases of RSV/SARS-CoV-2, two of RSV/influenza, and one of SARS-CoV-2/influenza, as presented in Table 4. Fisher’s exact test showed no significant association between co-infection and demographic factors (*p* = 0.62).

**Table 4:** Co-infection patterns

Co-Infection Type	Number of Cases	Percentage (%)
RSV/SARS-CoV-2	2	0.34
RSV/Influenza	2	0.34
SARS-CoV-2/Influenza	1	0.17
Total	5	0.9

† *Abbreviations*: *RSV* respiratory syncytial virus, *SARS-CoV-2* severe acute respiratory syndrome coronavirus 2. Co-infections were detected using multiplex real-time PCR. No significant association was found between co-infection and demographic factors (Fisher’s exact test, *p* = 0.62)

Virus positivity by sex showed no significant differences, as presented in Table 5. Influenza was detected in 25.1% of males (*n* = 92; 95% CI: 20.8–29.8) and 30.0% of females (*n* = 66; 95% CI: 24.0–36.5) (χ² = 1.91, *p* = 0.17). RSV was detected in 7.1% of males (*n* = 26; 95% CI: 4.7–10.2) and 4.1% of females (*n* = 9; 95% CI: 1.9–7.7) (χ² = 1.72, *p* = 0.19). SARS-CoV-2 was identified in 6.8% of males (*n* = 25; 95% CI: 4.4–10.0) and 5.5% of females (*n* = 12; 95% CI: 2.8–9.5) (χ² = 0.70, *p* = 0.40). Logistic regression, adjusting for age and region, confirmed no significant sex-based associations for influenza (adjusted OR: 1.28, 95% CI: 0.87–1.89, *p* = 0.21), RSV (adjusted OR: 1.77, 95% CI: 0.85–3.67, *p* = 0.13), or SARS-CoV-2 (adjusted OR: 1.24, 95% CI: 0.59–2.61, *p* = 0.57).

**Table 5:** Virus positivity by sex

Virus	Sex	Number Positive	Percentage (95% CI)	χ²	*p*-value
Influenza	Male	92	25.1% (20.8–29.8)	1.91	0.17
	Female	66	30.0% (24.0–36.5)		
RSV	Male	26	7.1% (4.7–10.2)	1.72	0.19
	Female	9	4.1% (1.9–7.7)		
SARS-CoV-2	Male	25	6.8% (4.4–10.0)	0.70	0.40
	Female	12	5.5% (2.8–9.5)		

† *Abbreviations*: *RSV* respiratory syncytial virus, *SARS-CoV-2* severe acute respiratory syndrome coronavirus 2; CI, confidence interval. Positivity rates were determined using multiplex real-time PCR. Statistical significance was assessed using chi-square tests (*p* < 0.05)

**Fig. 2:**
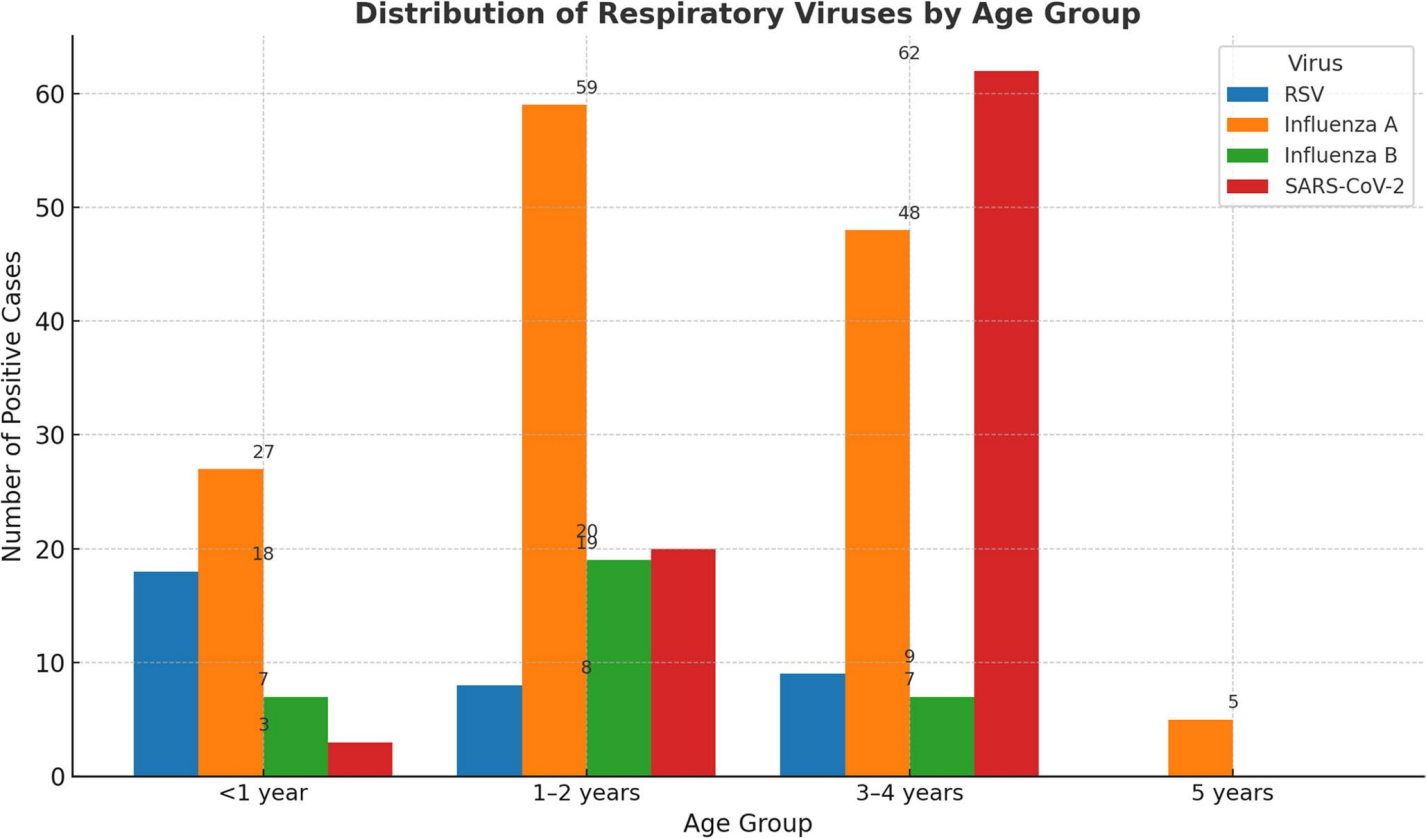
Age-specific distribution of respiratory viruses among hospitalized children <5 years, Hamedan Province, Iran, April 2023–March 2024. RSV predominated in infants <12 months, influenza A was most frequent in older children (24–59 months), and SARS-CoV-2 was distributed relatively evenly across all age groups

Age-stratified analysis revealed distinct patterns across the three respiratory viruses (Table 6; Fig. 2). RSV was most frequently detected among infants younger than 12 months (32.7%), consistent with the well-recognized vulnerability of this age group. In contrast, influenza A predominated in older children, accounting for 38.1% of detections in those aged 12–23 months and increasing to more than half of cases (55.7%) in the 3–4-year age group. SARS-CoV-2 demonstrated a relatively even distribution across all ages, with the highest frequency observed among children aged 3–4 years (49.2%). Influenza B was less frequently detected overall, contributing modestly across all age strata.

**Table 6:** Virus positivity by age

Age group	RSV *n* (%)	Influenza A *n* (%)	Influenza B *n* (%)	SARS-CoV-2 *n* (%)	Total *n*	
< 1 year	18 (32.7%)	27 (49.1%)	7 (12.7%)	3 (5.5%)		55
1–2 years	8 (7.6%)	59 (55.7%)	19 (17.9%)	20 (18.9%)		106
3–4 years	9 (7.1%)	48 (38.1%)	7 (5.6%)	62 (49.2%)		126
5 years	0 (0.0%)	5 (100.0%)	0 (0.0%)	0 (0.0%)		5
Total	35 (6.0%)	139 (23.7%)	33 (5.6%)	85 (14.5%)		230

† The table summarizes the number and percentage of RSV, influenza A/B, and SARS-CoV-2 cases detected in predefined pediatric age groups (<12 months, 12–23 months, 24–59 months). Data are reported descriptively to illustrate age-related patterns of virus circulation. *Percentages are calculated relative to the total number of positive cases within each age group, not the entire study population*

**Fig. 3:**
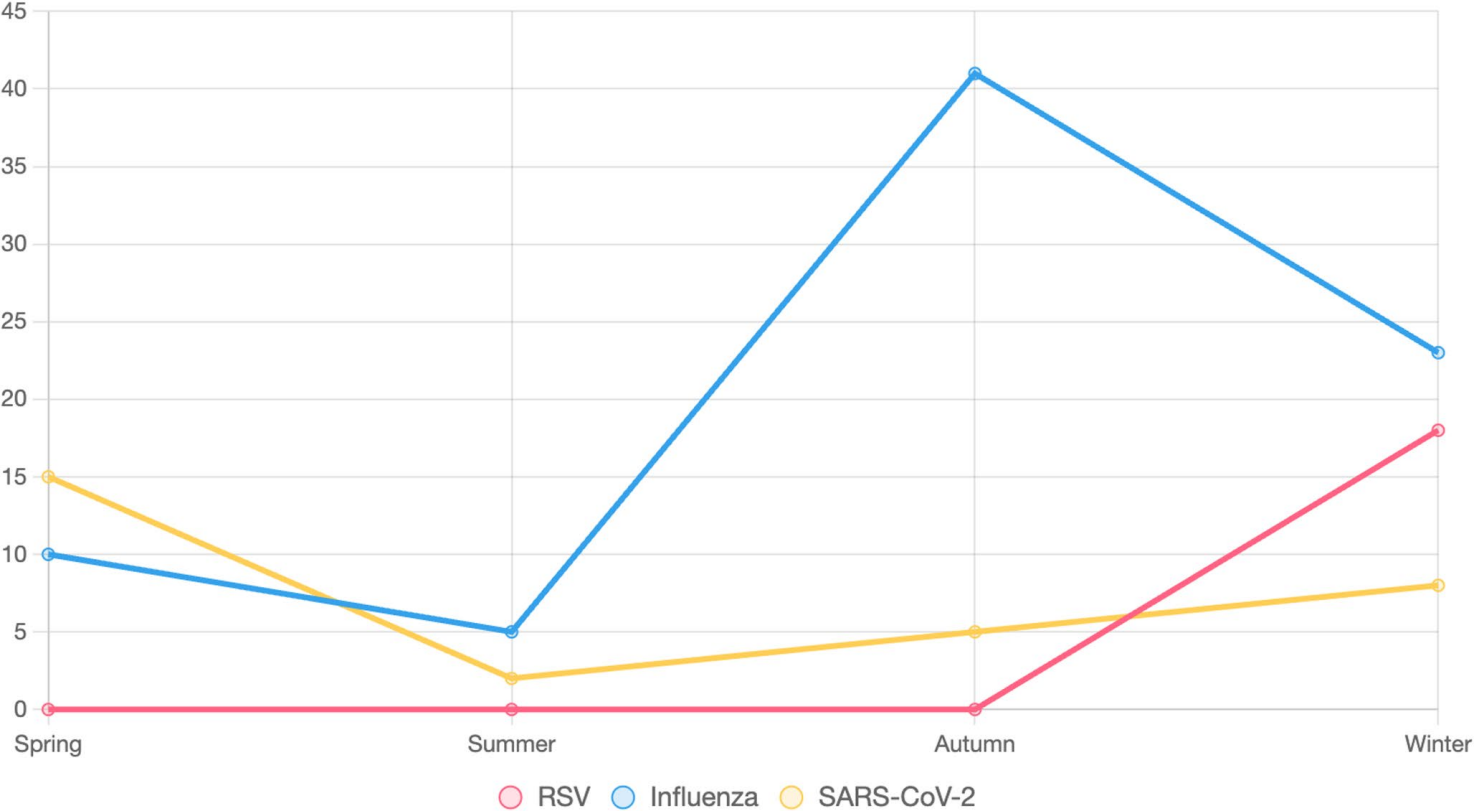
Seasonal distribution of respiratory virus positivity in children aged 0–5 Years, Hamedan Province, Iran (2023–2024). Line plots depict the percentage of RSV, influenza, and SARS-CoV-2 cases detected in each season, analyzed using generalized linear models (GLMs) with a binomial distribution and logit link (p < 0.01 for all viruses). Influenza peaked in autumn (41.3%), RSV in winter (18.2%), and SARS-CoV-2 in spring (15.3%), indicating distinct seasonal circulation patterns

Seasonal analysis revealed distinct temporal distributions across viruses, as illustrated in Fig. 3. RSV was detected exclusively in winter (*n* = 35; 18.2%), with no cases in other seasons (χ² = 13.8, df = 3, *p* < 0.001). Influenza peaked in autumn (*n* = 110; 41.3%), followed by winter (*n* = 43; 23.0%) (χ² = 35.6, df = 3, *p* < 0.001). SARS-CoV-2 exhibited a spring peak (*n* = 17; 15.3%), with lower rates in other seasons (χ² = 12.8, df = 3, *p* = 0.005). Generalized linear models with binomial distribution and logit link confirmed significant seasonal variation for all viruses (*p* < 0.01).

## Discussion

The COVID-19 pandemic, through widespread non-pharmaceutical interventions (NPIs), profoundly altered the epidemiology of pediatric respiratory viruses, disrupting their seasonality and co-circulation patterns [20, 21]. This province-wide surveillance study in Hamedan, a semi-arid region representative of western Iran, conducted from April 2023 to March 2024, provides critical insights into the post-pandemic dynamics of respiratory syncytial virus (RSV), influenza A and B, and SARS-CoV-2 among hospitalized children aged 0–5 years. As the first molecular surveillance effort of its kind in western Iran, this study addresses a significant data gap in the Middle East, where regional factors such as climate and healthcare access uniquely shape viral transmission [17]. Three key findings emerged: influenza re-established dominance as the primary viral pathogen, RSV exhibited suppressed circulation limited to winter, and SARS-CoV-2 displayed an atypical spring peak, reflecting ecological and immunological shifts in a vulnerable pediatric population.

Influenza was detected in 27.0% of hospitalized cases (*n* = 158, 95% CI: 23.4–30.8), with a pronounced peak in autumn (41.3%, *n* = 110/264, *p* < 0.001) and sustained circulation into winter (23.0%, *n* = 43/187), as shown in Fig. 1. This resurgence aligns with global trends observed after the relaxation of NPIs, as reported in Australia (winter 2022 surge) and Canada (autumn 2023 peak) [22, 23]. The concurrent circulation of influenza A subtypes (H1N1: 35%, H3N2: 25%) and influenza B (40%) underscores a high viral diversity, likely driven by reduced population immunity following limited exposure during the pandemic [24]. Notably, influenza positivity was higher among females (30.0% vs. 25.1%, *p* = 0.17), which may reflect sex-based immunological differences or differential exposure patterns, as seen in prior pediatric contact studies in Thailand and elsewhere [24]. Hamedan’s semi-arid climate, characterized by cold winters and dry conditions, may have amplified autumn transmission, as low humidity facilitates aerosolized viral spread [17]. The substantial clinical burden in this inpatient cohort emphasizes the urgent need for high influenza vaccination coverage, with campaigns prioritized for early autumn to preempt seasonal peaks in resource-limited settings.

RSV circulation was markedly reduced, detected in only 6.0% of cases (*n* = 35, 95% CI: 4.2–8.2) and confined exclusively to winter (18.2%, *n* = 35/187, *p* < 0.001). This contrasts sharply with pre-pandemic RSV incidence in Iran, which ranged from 16% to 22% with broader seasonality (autumn to spring) [25, 26]. The suppression may stem from multiple factors: residual immunity gaps from decreased exposure during NPIs, sustained behavioral changes (e.g., delayed daycare attendance, improved hygiene), and ecological competition from influenza’s rapid resurgence. In contrast, other regions have reported intense RSV rebounds post-COVID. For example, southern Brazil recorded nearly 3,000 RSV cases in a sharp 2021 resurgence, driven by delayed seasonality [27], while Sydney’s 2022 winter surge saw elevated hospitalization rates among RSV-infected infants, despite no variant-driven changes [28]. Hamedan’s cold, semi-arid climate may modulate RSV’s winter confinement, as low temperatures favor its stability [17]. Given RSV’s historical role as a leading cause of pediatric hospitalization globally, ongoing surveillance is critical to anticipate potential future surges, particularly in regions with limited RSV prophylaxis access.

SARS-CoV-2 was identified in 6.3% of cases (*n* = 37, 95% CI: 4.5–8.6), with an unexpected spring peak (15.3%, *n* = 17/111, *p* = 0.005). This deviates from its traditional winter dominance, reflecting a decoupling likely driven by variant evolution and hybrid immune landscapes [29, 30]. Multi-region studies, including those in China and New Zealand, have similarly reported spring or summer SARS-CoV-2 surges, attributed to variant-specific transmissibility and waning immunity rather than climatic factors alone [31]. In Hamedan, the spring signal may indicate waning maternal antibodies, low pediatric vaccine uptake, or regional re-exposure patterns among young children. The absence of genomic sequencing limits our ability to confirm variant-specific drivers, such as Omicron sublineages, which have been linked to altered seasonality globally [30]. These findings underscore the need for continuous SARS-CoV-2 monitoring, particularly in spring, to guide pediatric vaccination strategies in resource-constrained settings.

The age-stratified distribution observed in our pupolation is consistent with previous reports showing RSV predominance in infants and influenza affecting older preschool-aged children. In contrast, SARS-CoV-2 showed a relatively uniform distribution across all age groups, underscoring the need for continued surveillance to clarify whether this pattern persists over time.

Co-infections were rare (0.9%, *n* = 5), significantly lower than global inpatient reports of up to 18% [32], as detailed in Table 5. This low rate may reflect temporal separation of viral peaks, with influenza dominating autumn, RSV in winter, and SARS-CoV-2 in spring, potentially coupled with innate immune interference [33]. For instance, the absence of concurrent RSV and influenza or SARS-CoV-2 infections supports antagonistic interactions, as noted in Lithuanian pediatric studies during 2021–2022 [34]. However, co-detection does not necessarily imply functional interference, and biological validation is needed. The inpatient focus of this study likely underestimates co-infections, which are more prevalent in milder outpatient settings, as reported in recent Chinese and Australian studies (e.g., up to 25% co-detection in community-acquired infections) [35, 36]. These differences highlight the importance of integrating outpatient data to capture broader transmission dynamics.

**Fig. 4:**
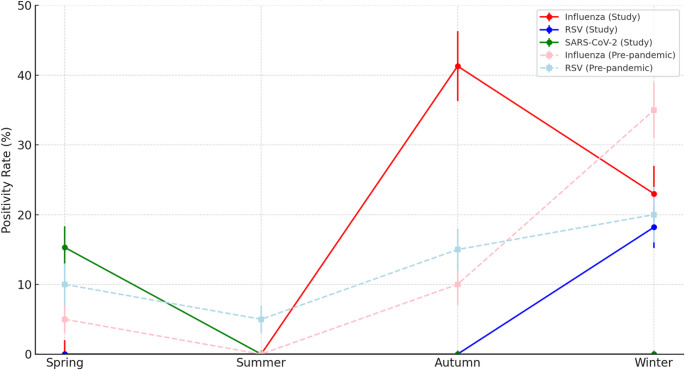
Seasonal distribution of respiratory viruses in hospitalized children: Hamadan 2023–2024 vs. pre-pandemic Iran. The figure compares intra-annual positivity rates of influenza, RSV, and SARS-CoV-2 in the present study (solid lines, 95% CI) with pre-pandemic Iranian data (dashed lines). Influenza showed an autumn peak (41.3%) instead of the typical winter dominance. RSV activity was reduced and confined to winter (18.2%), contrasting with its broader pre-pandemic seasonality. SARS-CoV-2 displayed a novel spring peak (15.3%), with no pre-pandemic counterpart. These findings illustrate altered post-COVID-19 seasonal dynamics

Our study was designed as a one-year cross-sectional surveillance, which allowed us to describe the intra-annual distribution of RSV, influenza A/B, and SARS-CoV-2 in hospitalized children during the post-COVID-19 era. Although our dataset does not permit direct inter-annual comparisons, placing our findings in the context of pre-pandemic reports from Iran highlights notable differences. For instance, earlier studies reported RSV peaks typically in late autumn and winter, while our results suggest a delayed or shifted circulation pattern. Similarly, influenza circulation, which was markedly suppressed during the COVID-19 pandemic, appeared to re-emerge in our cohort (Fig. 4). These observations should be interpreted as indirect evidence of altered seasonality in the post-COVID period, rather than as a direct measurement of multi-year changes. Long-term, multi-year surveillance will be essential to confirm whether these apparent shifts persist over time.

The observed patterns likely result from a complex interplay of immunological, ecological, and behavioral factors. RSV’s suppressed reappearance may reflect an “immunity debt” from reduced exposure during NPIs, compounded by influenza’s ecological dominance [37, 38]. Similarly, SARS-CoV-2’s spring surge suggests adaptation to shifting host immunity, potentially exacerbated by low vaccination coverage in Iranian children. Hamedan’s semi-arid climate, with low humidity enhancing influenza transmission and cold winters favoring RSV stability, further modulates these dynamics [17]. These findings can inform Iran’s national vaccination programs, emphasizing early autumn influenza vaccination and winter RSV prophylaxis to mitigate household transmission in resource-limited settings.

This study provides valuable insights but is subject to certain limitations. The focus on an inpatient population with severe cases offers a critical perspective on pediatric disease burden but may limit generalizability to community settings, potentially overestimating severe case incidence and underestimating community-level co-infections. A key limitation is the low rate of co-infections, likely due to restricting diagnostics to SARS-CoV-2, influenza A/B, and RSV. This inevitably underestimates the true burden, as previous studies using multiplex panels that detect additional viruses (e.g., HRV/EV, AdV, PIV, hMPV) have reported pediatric co-infection rates up to 30–40%. Children under five are particularly prone to mixed infections owing to immature immunity and communal exposures. Although SARS-CoV-2/influenza co-infections remain rare (2–16%), they are linked to worse outcomes [24, 39]. The absence of systematic clinical data further limited correlation of the few co-infections with symptom profiles, constraining insights into their prognostic significance.

The absence of genomic sequencing restricts insights into variant-specific seasonality, and the lack of clinical severity scoring precludes detailed outcome comparisons. Future studies could enhance understanding of these evolving patterns by incorporating whole-genome sequencing, longitudinal tracking, and combined outpatient-inpatient surveillance. As previously noted, the lack of systematic collection of clinical and symptomatic data limited our capacity to explore associations between viral detection, co-infections, and clinical outcomes.The low frequency of co-infections observed in our pupolation is also likely attributable to the fact that testing was restricted to three viruses (SARS-CoV-2, influenza, RSV), whereas higher co-infection rates are typically reported in children when broader viral panels are employed. Our analyses were therefore restricted to demographic variables, and detailed clinical correlations could not be made. Moreover, we did not capture socio-environmental determinants such as breastfeeding history, nutritional status, or housing conditions, which may also influence susceptibility to pediatric respiratory infections. Vaccination status was likewise not included, as RSV vaccination is not available in Iran, influenza vaccination is not part of the national pediatric immunization program and is rarely used voluntarily by parents, and COVID-19 vaccination is only authorized for children older than five years, while our study population comprised exclusively children under five. Future studies should integrate standardized clinical data, vaccination status, and multiplex molecular testing to better elucidate the epidemiology and clinical impact of single versus multiple infections in children. Despite these limitations, this study provides robust evidence for informing regional health policies.

From a policy perspective, these findings necessitate urgent recalibration of public health strategies. Sentinel hospital systems in Iran should prioritize real-time monitoring to detect seasonality shifts and emerging variants. Vaccination campaigns must align with observed trends: influenza vaccines should be administered in early autumn, RSV prophylaxis targeted for high-risk infants in winter, and SARS-CoV-2 monitoring extended into spring. Consideration of viral interference could guide intervention timing, prioritizing dominant pathogens seasonally. In resource-limited settings like Hamedan, where pediatric populations drive household transmission, a data-driven surveillance framework integrating clinical, molecular, and ecological data is essential for effective respiratory virus preparedness in the post-pandemic era.

## Conclusion

This study presents the first province-wide molecular surveillance of RSV, influenza, and SARS-CoV-2 in young children in post-pandemic Iran. The results highlight a shift in seasonal virus dynamics, with influenza regaining dominance, RSV activity restricted to winter, and an atypical spring peak in SARS-CoV-2. The low co-infection rate suggests competitive interactions between viruses in the pediatric population. These observations have critical implications for regional health policy, including the timing of vaccinations and the design of surveillance systems. Ongoing, adaptive monitoring of respiratory pathogens will be essential to mitigate future outbreaks and protect vulnerable pediatric populations in the evolving post-COVID landscape.

## Data Availability

The datasets generated and analyzed during this study are available from the corresponding author upon reasonable request, subject to ethical and privacy restrictions due to the involvement of pediatric patient data.

## Abbreviations

ARI: Acute Respiratory Infection
LMIC: Low- and Middle-Income Country
NPI: Non-Pharmaceutical Intervention
RSV: Respiratory Syncytial Virus
SARS-CoV-2: Severe Acute Respiratory Syndrome Coronavirus 2
PCR: Polymerase Chain Reaction
GLM: Generalized Linear Model
Ct: Cycle Threshold
VTM: Viral Transport Medium
MIS-C: Multisystem Inflammatory Syndrome in Children
CI: Confidence Interval
OR: Odds Ratio
SD: Standard Deviation
WHO: World Health Organization
InfA: Influenza A
InfB: Influenza B
SW: Swine Influenza A (H1N1)-related genes
H1: Influenza A subtype H1N1
AH3: Influenza A subtype H3N2
HA: Hemagglutinin
NA: Neuraminidase
